# Efficacy of Probiotic Supplementation with Lactobacillus and Bifidobacterium in Major Depressive Disorder: A Meta-analysis of Randomized Controlled Trials

**DOI:** 10.1192/j.eurpsy.2026.10605

**Published:** 2026-07-31

**Authors:** K. M. Henry Moore, N. M. Neves, G. Stutz Francisco Moreira, A. Mert, J. Rodrigues Lopes, D. Soler Lopes, T. Pereira Mendes

**Affiliations:** 1 Fundación H. A. Barceló, Buenos Aires, Argentina; 2 Faculdade de Medicina Santa Marcelina, Sao Paulo; 3 Federal Hospital of Servants of Rio de Janeiro, Rheumatology Department, Rio de Janeiro, Brazil; 4 Nazilli State Hospital, Psychiatry Department, Aydin, Türkiye; 5 Faculdade de Ciências Médicas de São José dos Campos- HUMANITAS, Sao Jose dos Campos, Brazil; 6 Lancaster University, UK, Lancaster, United Kingdom; 7 Department of Psychiatry and Legal Medicine, Institute of Psychiatry, Federal University of Rio de Janeiro, Rio de Janeiro, Brazil

## Abstract

**Introduction:**

Major depressive disorder (MDD) is a leading cause of global disability, often resistant to standard treatments. Increasing evidence implicates the gut–brain axis in its pathophysiology. Probiotics, especially *Lactobacillus* and *Bifidobacterium* strains, may exert antidepressant effects by modulating inflammation, neurotransmission, and the hypothalamic–pituitary–adrenal axis through their interaction with the gut microbiota.

**Objectives:**

This systematic review and meta-analysis of randomized controlled trials (RCTs) aimed to evaluate the efficacy of *Lactobacillus*- and *Bifidobacterium*-based probiotic supplementation in reducing depressive symptoms in individuals with MDD.

**Methods:**

We searched PubMed, Embase, and the Cochrane Library for RCTs comparing probiotic supplementation to placebo or standard therapy in adults with MDD. Included studies used validated depression scales such as the Hamilton Depression Rating Scale (HAMD), Beck Depression Inventory (BDI), or Depression Anxiety Stress Scales (DASS). The primary outcome was change in depressive symptom severity. A random-effects model (Hartung-Knapp-Sidik-Jonkman method) was used to compute standardized mean differences (SMDs) with 95% confidence intervals (CIs). Exploratory subgroup analyses were conducted based on probiotic formulation, dosage, and strain composition.

**Results:**

Eight RCTs with 539 participants were included. Of these, 279 patients (51.8%) received probiotic supplementation and 260 (48.2%) received placebo or standard therapy alone. Probiotic use was associated with a greater, though non-significant, reduction in depressive symptoms (SMD –0.34; 95% CI: –0.76 to 0.09; I² = 75%; Image 1). Subgroup analysis suggested larger effects with multistrain formulations and capsule-based delivery.

**Image 1:**
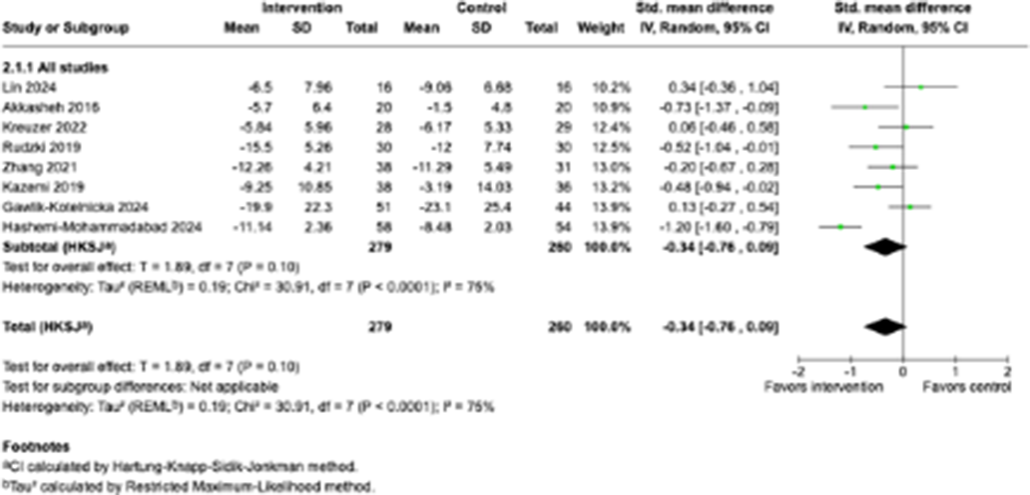
Image 1: Long description.

**Conclusions:**

Probiotic supplementation with *Lactobacillus* and *Bifidobacterium* may modestly reduce depressive symptoms in MDD. Subgroup trends favor multistrain, capsule-based products. Given their safety, low cost, and accessibility, probiotics represent a promising adjunctive strategy. Further trials are warranted.

**Disclosure of Interest:**

None Declared

## Long descriptions

### Image 1: Long description

A forest plot table with columns for Study or Subgroup, Intervention (Mean, S D, Total), Control (Mean, S D, Total), Weight, and Std. mean difference I V, Random, 95% C I.

Individual study data:

* Lin 2024: 0.34 [-0.36, 1.04]

* Akkasheh 2016: -0.73 [-1.37, -0.09]

* Kreuzer 2022: 0.06 [-0.46, 0.58]

* Rudzki 2019: -0.52 [-1.04, -0.01]

* Zhang 2021: -0.20 [-0.67, 0.28]

* Kazemi 2019: -0.48 [-0.94, -0.02]

* Gawlik-Kotelnicka 2024: 0.13 [-0.27, 0.54]

* Hashemi-Mohammadabad 2024: -1.20 [-1.60, -0.79]

The Subtotal and Total (H K S J super a) for 279 intervention and 260 control participants is -0.34 [-0.76, 0.09].

On the right, the forest plot shows a vertical line at 0. Most study markers (green squares) and their horizontal error bars lie to the left of the center line, favoring intervention. A black diamond at the bottom represents the pooled effect, centered at -0.34, touching the zero line.

Statistical notes:

* Heterogeneity: Tau super 2 (R E M L super b) = 0.19; Chi super 2 = 30.91, d f = 7 (P < 0.0001); I super 2 = 75%.

* Test for overall effect: T = 1.89, d f = 7 (P = 0.10).

Navigate back to Image 1:.

